# Dihydroquercetin Attenuates Silica-Induced Pulmonary Fibrosis by Inhibiting Ferroptosis Signaling Pathway

**DOI:** 10.3389/fphar.2022.845600

**Published:** 2022-05-12

**Authors:** Leyong Yuan, Yan Sun, Ning Zhou, Weipeng Wu, Weidong Zheng, Yukun Wang

**Affiliations:** ^1^ Department of Clinical Laboratory, Southern University of Science and Technology Hospital, Shenzhen, China; ^2^ Hubei Key Laboratory of Wudang Local Chinese Medicine Research, Hubei University of Medicine, Shiyan, China; ^3^ Department of Laboratory Medicine, Shenzhen University General Hospital, Shenzhen, China; ^4^ Department of Pharmacology, School of Medicine, Southern University of Science and Technology, Shenzhen, China

**Keywords:** silicosis, dihydroquercetin, lung fibrosis, ferroptosis, ferritinophagy

## Abstract

Silicosis is a fatal occupational lung disease which currently has no effective treatment. Dihydroquercetin (DHQ) is a flavonoid compound known for its anti-inflammatory, anti-oxidant and anti-cancer bioactivity. However, whether DHQ protects against silica-induced lung fibrosis remains unknown. Therefore, we aimed to investigate the effect of DHQ on silica-induced lung fibrosis and the underlying molecular mechanism *in vivo* and *in vitro*. Our results demonstrated that DHQ treatment markedly attenuated SiO_2_-induced inflammation and fibrosis degree of lung tissues in the C57BL/6 mice. Additionally, experiments *in vitro* also confirmed that conditioned medium from DHQ-treated human bronchial epithelial (HBE) cells significantly decreased expression of fibrosis markers of human fetal lung fibroblast cells (MRC-5), such as α-SMA, collagen1 and fibronectin. Interestingly, HBE cells treated by DHQ showed few morphological features of ferroptosis compared with SiO_2_-treated cells. Furthermore, DHQ treatment remarkably inhibited ferroptosis in activated HBE cells by decreasing the accumulation of iron and lipid peroxidation products, and increasing levels of glutathione (GSH) and glutathione peroxidase 4 (GPX4), whereas stimulation of ferroptosis by specific inducer erastin deeply impaired anti-fibrosis effect of DHQ *in vitro*. More importantly, our results showed that DHQ also evidently suppressed ferritinophagy by down-regulation of microtubule-associated protein 1A/1B-light chain 3 (LC3), and up-regulation of ferritin heavy chain 1 (FTH1), nuclear receptor co-activator 4 (NCOA4) in activated HBE cells. Nevertheless, activation of ferritinophagy by specific inducer rapamycin (Rapa) evidently blocked DHQ-inhibited HBE cells ferritinophagy and anti-fibrosis effect of DHQ. Overall, our research revealed that inhibition of ferritinophagy-mediated HBE cells ferroptosis was responsible for DHQ to ameliorate SiO_2_-induced lung fibrosis, which provided a preliminary theoretical basis for the clinical application of DHQ in the treatment of silicosis.

## Introduction

Silicosis is an occupational lung disease caused by chronic exposure to silica (SiO_2_) dust, which is characterized by persistent inflammation and progressive fibrosis in lung tissue (Leung et al., 2012). The inflammation cell infiltration, hyperproliferation of fibroblasts, and excessive deposition of extracellular matrix (ECM) contribute to the development of silicosis (Fang et al., 2018). The incidence and prevalence of silicosis, despite continuous efforts geared at improving the work environment, continues to rise worldwide, especially in developing countries (Casey and Mazurek, 2019; Hoy and Chambers, 2020). However, the underlying molecular mechanisms of silicosis are poorly understood, and there is currently no effective treatment to block progression of lung fibrosis or reverse it. Therefore, there is yet a critical requirement to develop more effective therapeutic strategies.

Dihydroquercetin (DHQ), also known as taxifolin, is a typical plant flavonoid found in yew, larch and cedrus brevifolia bark. Increasing studies showed that DHQ has anti-oxidant, anti-inflammatory, anti-proliferation, anti-radiation, anti-tumor, anti-viral pharmacological activities and anti-anaphylaxis effects (Zhang et al., 2017; Ding et al., 2018; Pan et al., 2019; Wang R. et al., 2020a; Gunesch et al., 2020; Ricke-Hoch et al., 2021). However, the protective effects of DHQ on silica-induced pulmonary fibrosis are unclear. Therefore, the objective of the present study is to determine the antifibrotic effects of DHQ on silicosis and to further explore its fundamental molecular mechanism.

Ferroptosis is a novel type of programmed cell death, that is, characterized by iron dependent accumulation of reactive oxygen species (ROS) and lipid peroxidation (Dixon et al., 2012). Previous studies revealed that ferroptosis performs a significant function in a variety of organic diseases, including lung, heart, brain, liver, and kidney diseases (Sarhan et al., 2018; Zhu and Sun, 2018; Weiland et al., 2019; Capelletti et al., 2020). Thus, targeting ferroptosis might offer a promising therapeutic strategy for these diseases. Until now, some mechanisms have been proven to participate in the regulation of ferroptosis containing suppression of cystine/glutamate (system Xc-), reduction of glutathione (GSH) level, and restraint of glutathione peroxidase 4 (GPX4) activity (Sun et al., 2016; Sarhan et al., 2018; Zhu and Sun, 2018; Capelletti et al., 2020). Remarkably, a large number of studies have indicated that ferroptosis is strongly correlated with autophagy (Hou et al., 2016; Hamai and Mehrpour, 2017; Park and Chung, 2019; Liu J. et al., 2020a). Autophagy is a catabolic cellular process that conservatively maintains cellular homeostasis and cellular survival under stress conditions, such as starvation or hypoxia (Ravanan et al., 2017; Kwon et al., 2018; Quaschling et al., 2020). Intriguingly, recent studies reported that an autophagic cargo receptor nuclear receptor coactivator 4 (NCOA4) delivers ferritin to autophagosomes for lysosomal degradation and iron release (Mancias et al., 2014; Gryzik et al., 2017). The latter process is referred to as ferritinophagy and is predominantly responsible for iron homeostasis (Mancias et al., 2014; Gao et al., 2016; Latunde-Dada, 2017; Masaldan et al., 2018; Zhang et al., 2018). More importantly, recent studies have discovered that ferritinophagy participates in the process of ferroptosis (Gao et al., 2016; Latunde-Dada, 2017; Masaldan et al., 2018; Zhang et al., 2018). However, the role of ferritinophagy in human bronchial epithelial (HBE) cells ferroptosis and silica-induced pulmonary fibrosis has not been studied.

In the present study, the effects of DHQ on lung fibrosis were investigated for the first time *in vitro* and *in vivo*, and the molecular mechanisms were further assessed. Our study confirmed that DHQ alleviated silica-induced lung fibrosis by inhibiting ferritinophagy mediated-ferroptosis. Our study provided an experimental basis for identifying ferroptosis as a potential therapeutic target for silicosis.

## Materials and Methods

### Reagents and Chemicals

Dihydroquercetin (DHQ) (Cat#480-18-2, purity above 98 percent) was purchased from Aladdin Biochemical Technology Co., Ltd. (Shanghai, China). Erastin (Cat#E7881) and rapamycin (Cat#53123-88-9) were purchased from Sigma Aldrich (Billerica, MA, United States). The SiO_2_ (Cat#S5631) particles (approximately 80% diameter: 1–5 μM) were purchased from Sigma-Aldrich and were filtered by sedimentation in accordance with the law of Stokes, subjected to acidic hydrolysis, and baked throughout the night (200°C, 16 h). The SiO_2_ particles were prepared for use in both *in vivo* and *in vitro* experiments. 3-(4,5-dimethylthiazol-2-yl)-2,5-diphenyl tetrazolium bromide (MTT) (#BS350B) as well as trypan blue exclusion assay kit (#BL707A) were obtained from Biosharp (Anhui, China). Antibodies against GPX4 (ab125066), ferritin heavy chain 1 (FTH1) (ab65080), NCOA4 (ab86707), and microtubule-related protein 1A/1B-light chain 3 (LC3) (ab192890) were all purchased from Abcam Technology (Abcam, Cambridge, United Kingdom). Antibodies against fibronectin (#26836), collagen I (#72026), alpha-smooth muscle actin (α-SMA) (#19245), and GAPDH (#5174), were acquired from Cell Signaling Technology Inc. (Danvers, MA, United States). A secondary antibody was obtained from Proteintech Biotechnology (Wuhan, China).

### Animals and Procedures

Eight-week-old male C57BL/6 mice were purchased from Hubei University of Medicine (Shiyan, China). The SiO_2_-induced mouse pulmonary fibrosis model was performed. In brief, each group of mice was anesthetized with 1% pentobarbital sodium intraperitoneally at 40 mg/kg body weight and their tracheae had been surgically exposed. In addition, SiO_2_ suspension (20 mg in 50 ul saline) was instilled in the mice. The vehicle control groups were given an equivalent amount of 0.9% sterile saline. After one week of acclimation, mice were divided randomly into four groups (*n* = 8 per group). Control group, SiO_2_ group, SiO_2_ and low dose of DHQ group (DHQ-L, 10 mg/kg) as well as large dose of DHQ group (DHQ-H, 50 mg/kg). One week later after SiO_2_ administration, DHQ (10 mg/kg or 50 mg/kg) was orally administered to mice at dosages of 10 mg/kg or 50 mg/kg once daily consecutively for 14 days. Meanwhile, the control and the SiO_2_ groups received an equal volum of 0.9% sterilized saline. On the day 21, mice were euthanized by intraperitoneal injection of an overdose of pentobarbital sodium. Blood samples from each group were collected for subsequent analysis. Lung tissues were excised and weighed, and the lung weight/body weight (LW/BW, mg/g) ratio was defined as the lung weight divided by the body weight. The left lower lobe lung samples were fixed in 10% formalin for histopathological analysis, and the remaining lung samples were stored at −80°C. All experiments were approved by the Institutional Animal Care and Use Committee of Hubei University of Medicine (Shiyan, China), and performed in accordance with the Provision and General Recommendation of the Chinese Experimental Animals Administration Legislation.

### Tissue Collection and Lung Histopathology

After the mice were sacrificed, lung tissues were collected quickly and fixed with 4% paraformaldehyde, dehydrated with alcohol gradient and then paraffin-embedded. Then the samples were cut into 5 μM sections and were stained for hematoxylin and eosin (H&E) or improved special masson trichrome. The pathological sections of lung tissue were observed and photographed under light microscope.

### Histological Scoring Evaluation

Sections were stained with hemotoxylin and eosin for the inflammation score evaluation and grading were classified according to the following criteria: none (0), no alveolitis; mild (1+), affected area<20%; moderate (2+), affected area ∼20–50%; severe (3+), affected area >50%. Fibrosis score was evaluated using masson’s trichrome stain sections. The severity of fibrosis in these sections was graded according to the following criteria: none (0), no evidence of fibrosis; mild (1+); lesion range of fibrosis<20% in the whole lung; moderate (2+), lesion range of fibrosis ∼20–50% in the whole lung; severe (3+), lesion range of fibrosis >50% in the whole lung.

### Hydroxyproline Assay

The hydroxyproline (HYP) contents in the lung tissues were determined using an HYP kit (#KGT030-2, KeyGen Biotech, Nanjing, China), according to the manufacturer’s instructions. Frozen lungs (∼100 mg) were cut into pieces and then homogenized on ice. Lung tissues were hydrolyzed in 1 ml lysis buffer at 100°C for 20 min. The mixture was incubated at 60°C for 15 min, cooled at room temperature and centrifuged at 8,000 g for 10 min. The absorbance was measured at 550 nm and the expression were calculated as µg hyp per mg wet lung weight.

### Enzyme-Linked Immunosorbent Assay

The levels of cytokines including interleukin-1β (IL-1β), tumor necrosis factor-α (TNF-α) and transforming growth factor-β (TGF-β) in serum and lung tissue homogenate were quantified by ELISA using the corresponding assay kit (#CSB-E08054m, #CSB-E04741m, #CSB-E04726m; Cusabio Biotech, Wuhan, China) following the manufacturer’s protocol. The optical density was measured at 450 nm for each cytokine. The minimum detection limit of IL-1β, TNF-α, and TGF-β was 7.8 pg/ml, 3.9 pg/ml and 0.2 ng/ml, respectively. The levels of the cytokines were obtained with a standard curve.

### Cell Culture

Human bronchial epithelial (HBE) cells and human fetal lung fibroblast MRC-5 cells were purchased from Procell (Wuhan, China) and cultured in DMEM supplemented with 10% fetal bovine serum and 1% streptomycin/penicillin mixture. When reaching about 80% confluence, cells were passaged with 0.25% trypsin digestion, and plated in 12-well plates and cultured overnight. Subsequently, the medium was changed to fresh culture medium with 10% fetal bovine serum, and subjected to different treatments. A humidified 5% CO2 atmosphere was used to maintain all cell cultures at 37°C.

### Treatment of MRC-5 Cells With Conditioned Media From SiO_2_ or Dihydroquercetin-Treated Human Bronchial Epithelial Cells

After overnight culture, HBE cells were incubated with SiO_2_ (50 ug per mL medium) or DHQ (40 μM) alone and co-treated with a mixture of SiO_2_, respectively. In the co-treatment, DHQ needs to act on HBE cells 1 h in advance and then mixed with SiO_2_. HBE cells in normal culture were used as the negative control. 24 h later, the obtained supernatant was collected and filtered through a 0.22 μM disposable filter. Meanwhile, the HBE cells were washed twice with phosphate-buffered saline (PBS) and protein was extracted for further experiments. The collected conditioned media were mixed with an equal amount of complete culture medium and were used to treat MRC-5 cells for 48 h. Then, the total protein was extracted and cell climbing tablets were made for later experiments.

### MTT Assay

MTT assay was conducted to assess cell viability according to the manufacturer’s instructions. HBE cells (1×10^5^ cells/well) were cultured in 96-well plates with serum-free DMEM medium for 24 h at 37°C in a 5% CO_2_ incubator. DHQ were diluted in PBS and then purified using a 0.22-µM pore size filter (Millex-GS; EMD Millipore). Then, the cells were incubated with 190 µl different concentrations of DHQ (0, 5, 10, 20, 40 or 80 µM). Following 24 h, the cells were incubated with 0.5% MTT at 37°C for 4 h. Subsequently, the supernatant was discarded and 150 µl dimethyl sulfoxide was added to each well for 10 min, and the plates were shaken on a horizontal shaker for 10 min. The absorbance of each sample was measured at 490 nm using a microplate reader.

### Trypan Blue Exclusion Assay

HBE cells were seeded in the 12 well culture plate at 1 × 10^5^ cells/well. When the cells of each well reached approximately 70% confluence, cells were exposed with SiO_2_ alone or in combination with DHQ (20 μM, 40μM, or 80 μM) for 48 h at 37°C with 5% CO_2_. Following treatment, HBE cells were trypsinized and collected by centrifugation at 700 × g for 5 min at room temperature. Cell suspension (100 μl) was mixed with equal volumes of 0.4% trypan blue for 5 min at room temperature. The viable cell were counted using a hemocytometer under an inverted microscope. Trypan blue stained the dead cells blue while the viable cells remained clear.

### Transmission Electron Microscopy

Transmission electron microscopy (TEM) was used to examine the mitochondrial features of HBE cells (H-7000, Hitachi, Japan). For intracellular structure observation, The cells were collected and fixed with 2.5% glutaraldehyde overnight, and the morphology of the HBE cells was observed by TEM. Quantification analysis of transmission electron microscopy was determined by the image J software. Any straight line on the image can be defined by two pixels on each end, pixel 1 and pixel 2. Therefore, coordinates of pixels were annotated *p*1=(*x*1, *y*1) and *p*2=(*x*2, *y*2) for pixels 1 and 2. The mitochondria length was measured by applying the pythagorean theorem to the coordinate pixels. Thus, where *Lp*1, *p*2 denotes the euclidean distance between pixel 1 and pixel 2. Length was given by (Equation: *L(p1+p2) = √(x1−x2)* ^*2*^ *+(y1−y2)* ^*2*^).

### Measurement of Iron, Glutathione, Reactive Oxygen Species, 4-Hydroxynonenal, and Malondialdehyde Level

The released iron, 4-HNE, and MDA level of HBE cell lysates were measured with iron, 4-HNE, and MDA assay kit (#ab83366, #ab118970, ab238538; Abcam) respectively following the directions of the manufacturer. The levels of ROS and GSH were assessed in HBE cells by the ROS assay kit (Beyotime, #S0033) and Glutathione assay kit (Beyotime, #S0053), and the guidelines stipulated by the manufacturer.

### Real-Time PCR Analysis

Trizol reagent (TAKARA, Japan) was utilized for the purpose of extracting total RNAs from the cell lines, and the PrimeScript™ RT Master Mix kit (TAKARA, Japan) was used to generate cDNAs from the total RNAs. The PCR was carried out utilizing the TB Green® Premix Ex Taq™ II kit (TAKARA, Japan) in accordance with the guidelines stipulated by the manufacturer. Supplementary Table S1 lists the primer sequences that were used in the present study. The conditions of PCR were shown as follows: 95°C for 5 min, then 40 cycles of denaturation at 95°C for 15 s, annealing 60°C for 30 s and extension at 72°C for 30 s. GAPDH served as the internal reference. The 2^−ΔΔCT^ method was used for calculation of the relative gene level.

### Western Blot Analysis

The protein levels of collagen I, fibronectin and α-SMA, GPX4, FTH1, NCOA4 and LC3 were detected by western blotting. Total proteins extracted from lung homogenate or cell lysate were lysed in RIPA buffer (Beyotime, #P0013B). Total protein concentration was determined by BCA protein assay kit (Beyotime, #P0012) and 50 µg protein was separated by SDS-PAGE gel, and transferred to a polyvinylidene difluoride (PVDF) membrane (Millipore, IPVH00010). The membrane was blocked for 2 h at room temperature in tris-buffered saline (TBS) (Sigma-aldrich, A9418), and then incubated with primary antibodies overnight at 4°C followed by detection with the HRP-labeled secondary antibodies and visualized using super ECL detection reagent (Beyotime, #P0018). The protein bands intensity was determined by the image J package and the relative protein levels were normalized to that of GAPDH.

### Immunofluorescence Analyses

For cell immunofluorescence, MRC-5 and HBE cells were fixed with 4% paraformaldehyde, washed with PBS, permeabilized with 0.2% Triton X-100 for 5 min at room temperature, and blocked with 1% bovine serum albumin for 1 h. MRC-5 and HBE cells were then incubated with primary antibodies against collagen-1, fibronectin, α-SMA, LC3 and GPX4 overnight at 4°C. After washing with PBS, cells were incubated with relative secondary antibody and counterstained with DAPI staining. The fluorescent intensity of target proteins was calculated using software image J.

### Statistical Analyses

All statistical analyses were performed by using the GraphPad 8.0.2 statistical software and results were presented as mean ± SD (standard deviation). Comparison among multiple groups were assessed by one-way ANOVA. Differences were defined as significant for *p* value < 0.05.

## Results

### Dihydroquercetin Protected Against SiO_2_-Induced Pulmonary Fibrosis in C57/BL6 Mice

**FIGURE 1:**
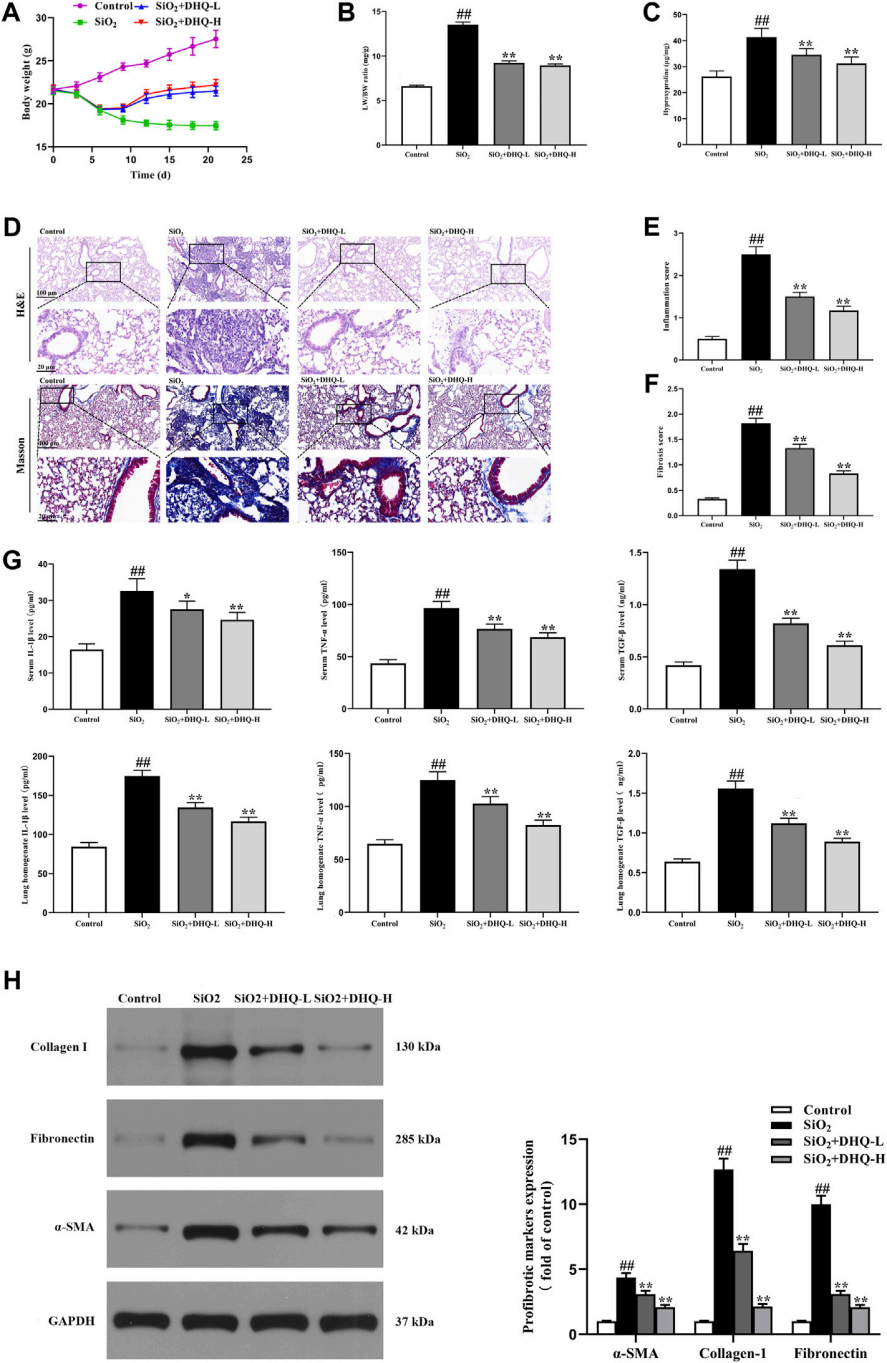
DHQ attenuated SiO_2_-induced pulmonary fibrosis in C57/BL6 mice One week after 0.2 g/kg SiO_2_ treatment, mice were orally administered with two doses of DHQ-L (10 mg/kg) and DHQ-H (50 mg/kg) once a day for 14 days. **(A)** The changes of body weight. **(B)** The changes of LW/BW ratio. **(C)** The HYP contents in lung tissues were determined. **(D)** Representative images ( × 200) of HE-stained and Masson-stained sections of mouse lung were shown. Bar = 100 μM. **(E)**, **(F)** The inflammation and fibrosis score numbers of 0–3, representing the grades of –, +, ++, and +++, were determined by experienced pathologists blindly. **(G)** The levels of pro-inflammatory cytokines in serum and lung homogenate were measured by ELISA. **(H)** The protein levels of α-SMA, collagen I and fibronectin in lung tissues were measured by western blotting. Data are shown as mean ± SD, All experiments were repeated three times. #*p* < 0.05, ##*p* < 0.01 vs. the control group; *p < 0.05, **p < 0.01 vs. the SiO_2_ group.

Two doses of DHQ-L (10 mg/kg) and DHQ-H (50 mg/kg) were orally administered for 14 consecutive days, respectively, starting 7 days after SiO_2_ (0.2 g/kg) administration. The high and low doses of DHQ treatment markedly attenuated SiO_2_-induced the weight loss and the increasing LW/BW ratio as well as HYP content in lungs (Figures 1A–C). HE and masson’s staining showed that DHQ ameliorated alveolar walls, inflammatory reaction, and deposition of collagen fibers at day 21 compared to SiO_2_ group (Figures 1D–F). Furthermore, the levels of pro-inflammatory cytokines, including IL-1β, TNF-α, and TGF-β, in serum and lung homogenate were elevated in SiO_2_ groups, but significantly reduced after DHQ treatment (Figure 1G). In addition, the expression of α-SMA, collagen I and fibronectin was also significantly reduced in DHQ-treated mice compared to SiO_2_ group (Figure 1H). Taken together, these findings further demonstrated that DHQ could greatly alleviate SiO_2_-induced inflammation and fibrosis degree of lung tissues.

### Dihydroquercetin Inhibited Profibrotic Markers in Fibroblasts

**FIGURE 2:**
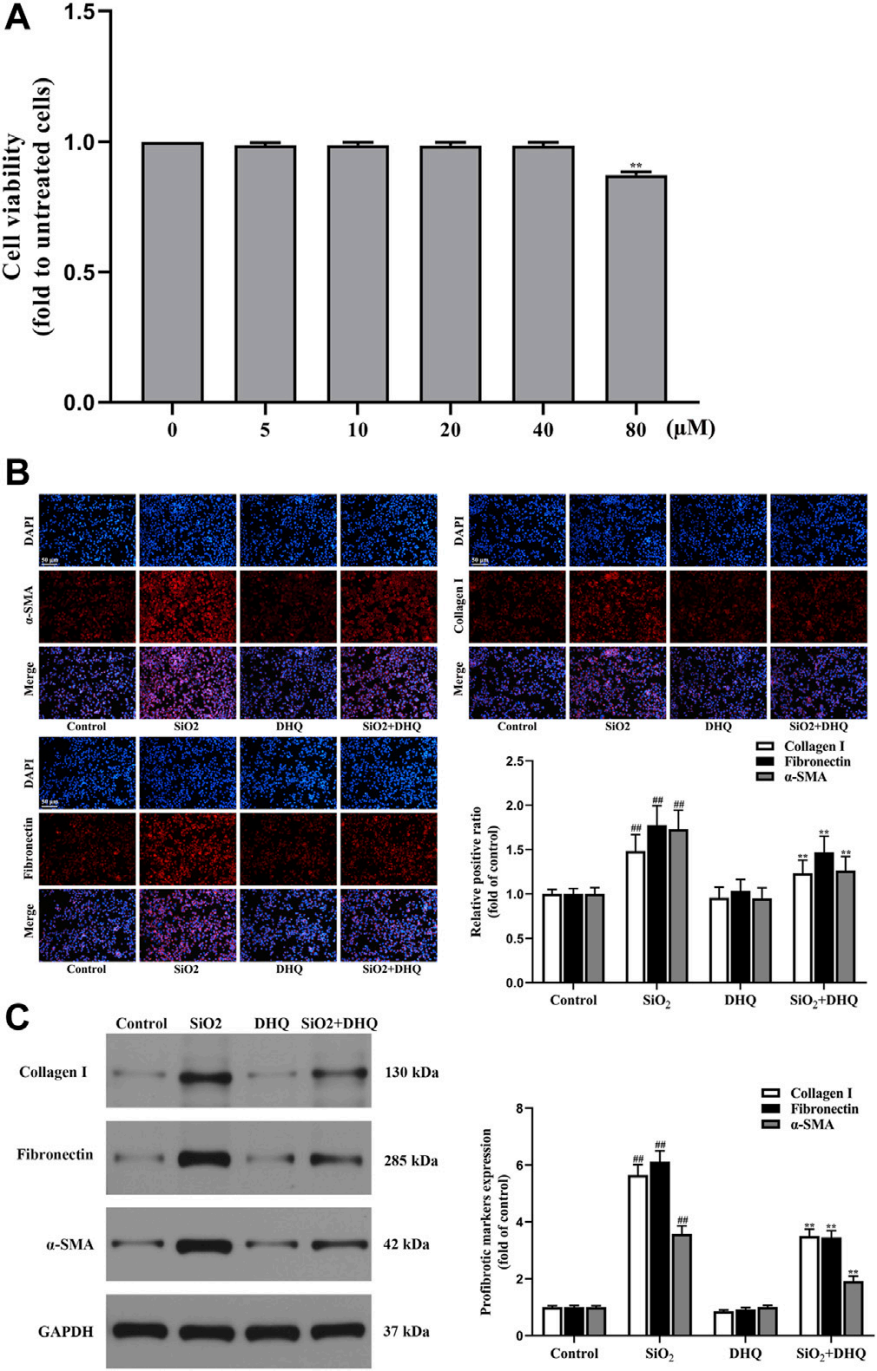
DHQ inhibited profibrotic markers in fibroblasts. **(A)** Effects of WEL (0–80 µM) on cell viability were determined by the MTT assays, **p < 0.01 vs. the 40 µM. **(B)** Representative images ( × 200) of immunofluorescence analysis of α-SMA, collagen I and fibronectin in MRC-5 cells. Bar = 50 μM. **(C)** The protein levels of α-SMA, collagen I and fibronectin in MRC-5 cells were measured by western blotting. D Data are shown as mean ± SD (*n* = 3). #*p* < 0.05, ##*p* < 0.01 vs. the control group; *p < 0.05, **p < 0.01 vs. the SiO_2_ group.

Our study had shown that DHQ attenuated SiO_2_-induced inflammation and pulmonary fibrosis *in vivo*. Subsequently, we conducted experiments *in vitro* to further corroborate experimental results *in vivo*. As demonstrated in Figure 2A, DHQ at concentration ranging from 5 to 40 µM had no obvious cytotoxicity to HBE cells. However, viability was below 90% when concentration reached 80 μM. Therefore, 40 μM of DHQ was used in our subsequent experiments *in vitro*. We used conditioned media from SiO_2_ or DHQ-treated HBE cells to verify whether DHQ treatment could inhibit MRC-5 cells activation *in vitro*. The immunofluorescence staining and western blot analysis of MRC-5 activation makers showed that DHQ significantly reduced the levels of α-SMA, collagen1 and fibronectin (Figures 2B–D). Altogether, conditioned media from DHQ-treated HBE cells inhibited MRC-5 cells activation.

### Dihydroquercetin Suppressed Ferroptosis of Activated Human Bronchial Epithelial Cells *in vivo* and *in vitro*

**FIGURE 3:**
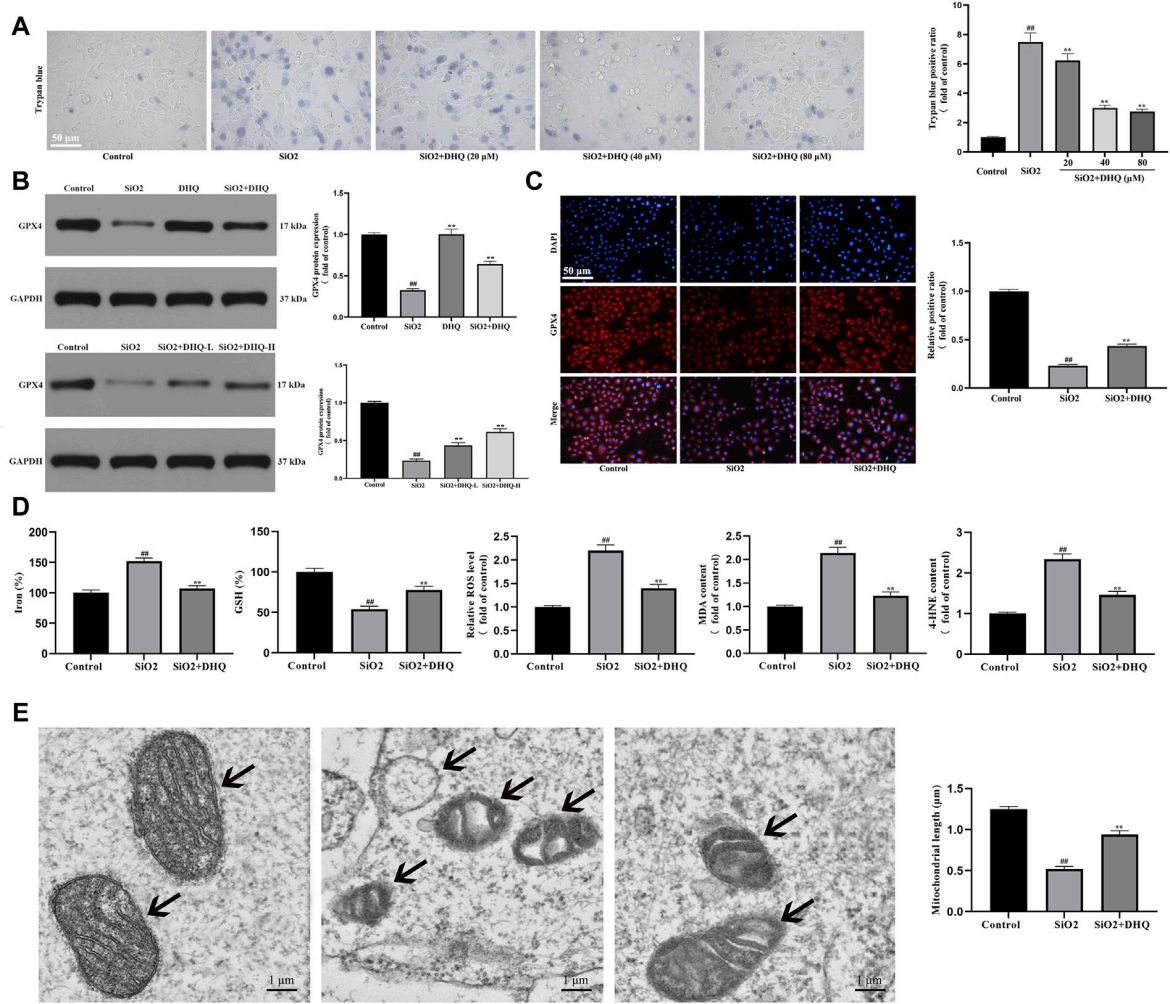
DHQ suppressed ferroptosis of activated HBE cells *in vivo* and *in vitro* **(A)** Representative images (✕200) of trypan blue staining for evaluating cell death. Bar = 50 μM. **(B)** The protein expressions of GPX4 in HBE cells and lung tissues were determined by western blotting. **(C)** Representative pictures ( × 200) of immunofluorescence analysis of GPX4 in HBE cells were determined by western blotting. Bar = 50 μM. **(D)** The levels of major biomarkers of ferroptosis (iron, GSH, ROS, MDA, and 4-HNE) in HBE cells were detected. **(E)** The morphological changes of mitochondria were observed by transmission electron microscopy and mitochondrial length was measured. Data are shown as mean ± SD (*n* = 3). #*p* < 0.05, ##*p* < 0.01 vs. the control group; *p < 0.05, **p < 0.01 vs. the SiO_2_ group.

Recently, ferroptosis was identified as an iron-dependent oxidative cell death that may be responsible for lung disorders (30-34). Our hypothesis in this study was that DHQ had a significant role in the anti-fibrosis effects by regulating ferroptosis. To validate this conjecture, we examined the effects of DHQ on the death levels of HBE cells. Trypan blue staining confirmed that the DHQ significantly reduced cell death in activated HBE cells (Figure 3A). Following that, we investigated whether DHQ mediated inhibition of the ferroptosis *in vivo* and *in vitro* by measuring ferroptosis-associated biomarkers. The results of immunofluorescence staining and western blot analysis displayed that the expressing of the ferroptosis marker GPX4 was significantly increased by DHQ treatment compared to SiO_2_ group (Figures 3B,C). Given that ferroptosis is mainly dependent on the intracellular iron accumulation, lipid peroxidation, and the depletion of antioxidant enzymes. The levels of Fe^2+^, ROS, MDA, 4-HNE content and GSH were detected to validate the occurrence of ferroptosis. As expected, our data also displayed that DHQ treatment markedly idecreased iron level and lipid peroxidation products, and increased GSH content (Figure 3D). Finally, electron morphology of cell mitochondria further testified the occurrence of ferroptosis. Compared with DHQ-treated HBE cells, SiO_2_-treated cells showed smaller, crumpled and broken mitochondria, which is cellular morphological characteristic of ferroptosis (Figure 3E). In conclusion, these data showed that DHQ suppressed activated HBE ferroptosis *in vivo* and *in vitro*.

### Activation of Ferroptosis Impaired the Anti-Fibrosis Effects of Dihydroquercetin *in vitro*

**FIGURE 4:**
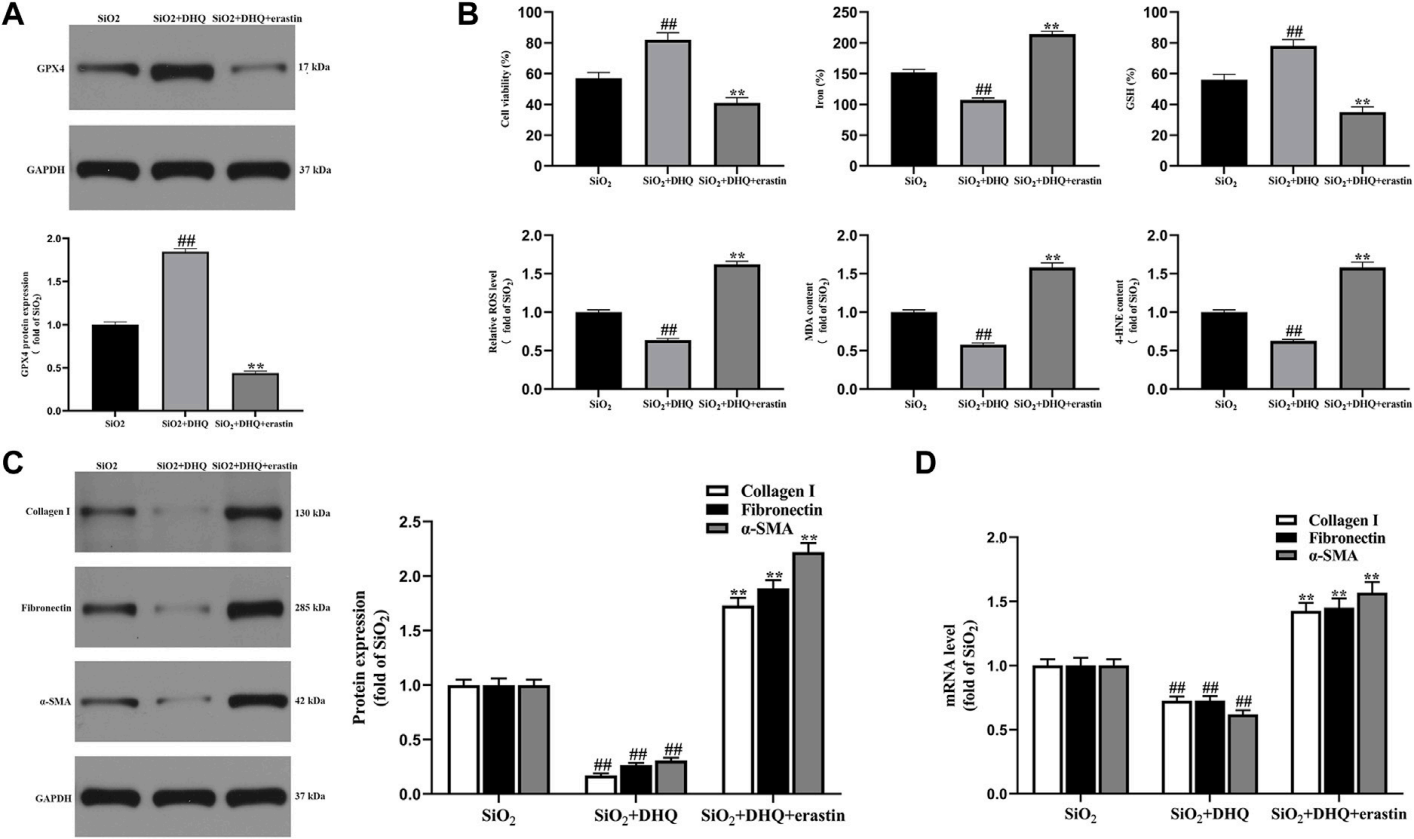
Activation of ferroptosis impaired DHQ-induced anti-fibrosis effects *in vitro* **(A)** The protein expressions of GPX4 in activated HBE cells were measured by western blotting. **(B)** The analysis of cell viability and major biomarkers of ferroptosis (iron, GSH, ROS, MDA and 4-HNE) in activated HBE cells. **(C)** The protein expressions of α-SMA, collagen I and fibronectin in MRC-5 cells stimulated with conditioned medium of HBE cells were measured by western blotting. **(D)** The analysis of real time-PCR of α-SMA, collagen I and fibronectin in MRC-5 cells. Data are shown as mean ± SD (*n* = 3). #*p* < 0.05, ##*p* < 0.01 vs. the SiO_2_ group; *p < 0.05, **p < 0.01 vs. the SiO_2_+DHQ group.

In order to further explore the role of ferroptosis in the anti-fibrotic effect of DHQ, ferroptosis specific inducer erastin was used to activate ferroptosis in activated HBE cells. As a result, HBE cells treated by DHQ exerted promotion of cell viability, whereas erastin showed the effect of cell survival inhibition (Figure 4B). In addition, DHQ treatment remarkably decreased the levels of iron, ROS, MDA and 4-HNE, but markedly increased the levels of GSH and GPX4 compared with SiO_2_ group. Interestingly, erastin treatment strongly blocked the inhibition effect of DHQ on ferroptosis (Figures 4A,B). Subsequently, real-time PCR assay and western blot analysis were carried out to determine whether stimulation of ferroptosis could reverse the inhibitory effect of DHQ on MRC-5 cells. Interestingly, the results indicated that treatment with DHQ significantly reduced the expression of α-SMA, collagen I and fibronectin, whereas this effect was significantly reversed by erastin (Figures 4C,D). These data collectively suggested that stimulation of ferroptosis impaired anti-fibrosis effects of DHQ *in vitro.*

### Dihydroquercetin Suppressed Ferroptosis in Activated HBE Cells *via* Inhibiting Ferritinophagy

**FIGURE 5:**
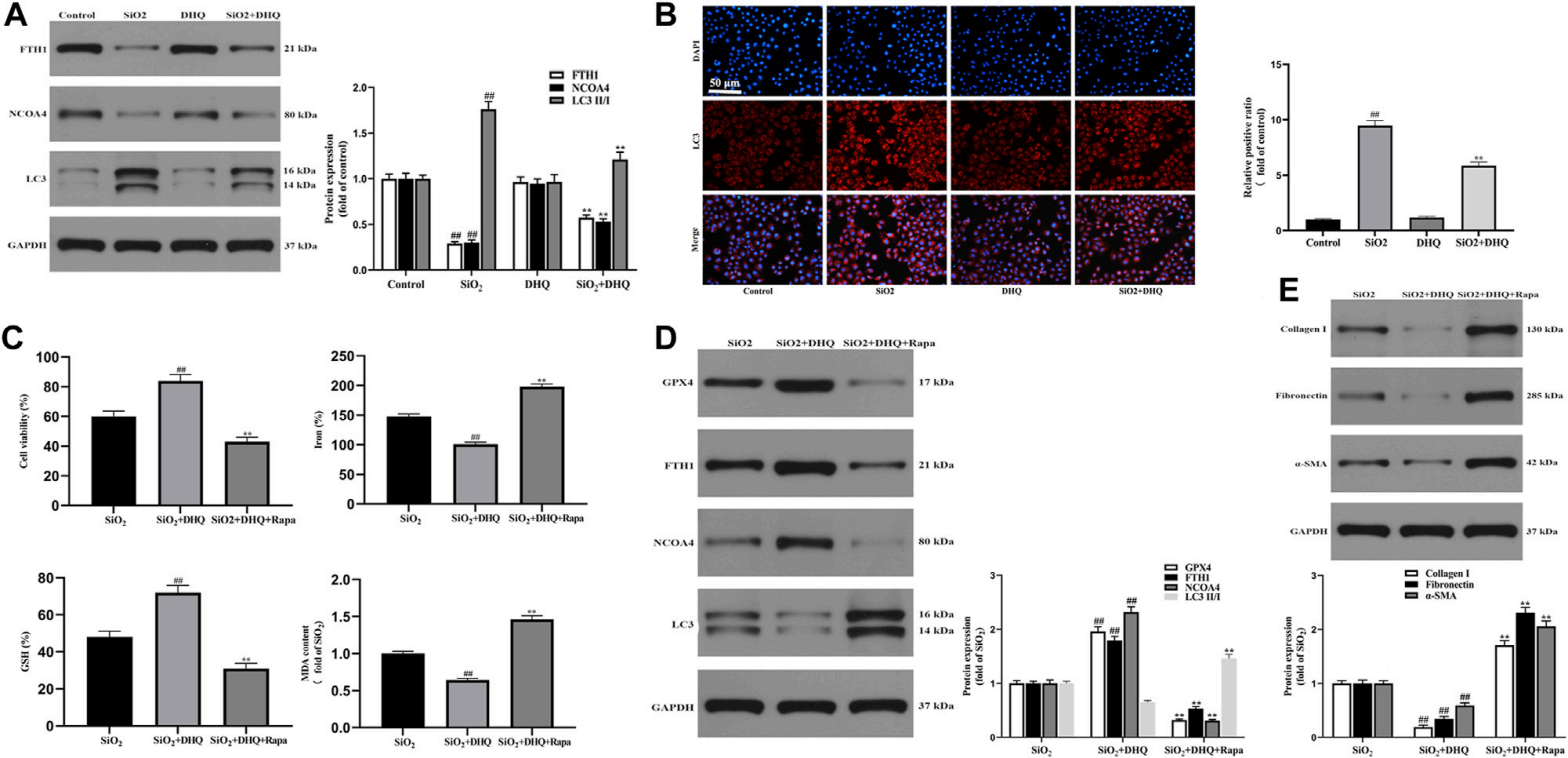
DHQ suppressed ferroptosis in activated HBE cells *via* inhibiting ferritinophagy. **(A)** The protein expressions of FTH1, NCOA4 and LC3 in activated HBE cells were determined by Western blotting. Data are shown as mean ± SD (*n* = 3). ##*p* < 0.01 vs. the control group; **p < 0.01 vs. the SiO_2_ group. **(B)** Representative pictures ( × 200) of immunofluorescence analysis of immunofluorescence analysis of LC3 in activated HBE cells. Bar = 50 μM. Data are shown as mean ± SD (*n* = 3). ##*p* < 0.01 vs. the control group; **p < 0.01 vs. the SiO_2_ group. **(C)** The analysis of cell viability and major biomarkers of ferroptosis (iron, GSH, ROS, MDA and 4-HNE) in activated HBE cells. **(D)** The protein expressions of GPX4, FTH1, NCOA4 and LC3 in activated HBE cells were measured by western blotting. **(E)** The protein expressions of α-SMA, collagen I and fibronectin in MRC-5 cells were determined by western blotting. Data are shown as mean ± SD (*n* = 3). ##*p* < 0.01 vs. the SiO_2_ group; **p < 0.01 vs. the SiO_2_+DHQ group.

An increasing number of studies have confirmed that the ferritinophagy participates in the occurrence and development of pulmonary diseases (Yoshida et al., 2019; Qin et al., 2021). Therefore, we speculated that DHQ ameliorated pulmonary fibrosis by regulating ferritinophagy of activated HBE cells. Western blotting revealed that the expression of vital autophagy makers LC3 was down-regulated, and the ferritinophagy markers FTH1 and NCOA4 were up-regulated by DHQ treatment (Figure 5A). Moreover, immunofluorescence staining displayed that DHQ inhibited the expression of LC3 (Figure 5B).

To further investigate the role of ferritinophagy in SiO_2_-induced pulmonary fibrosis, activated HBE cells were pretreated with rapamycin (Rapa), followed by DHQ treatment for 24 h. Our results demonstrated that DHQ alleviated ferroptosis by promoting cell viability, decreasing Fe^2+^ level and MDA content, and increasing GSH content, which was reversed by Rapa co-treatment (Figure 5C). In addition, results from western blot showed that DHQ treatment significantly increased the level of GPX4 and decreased the expression of ferritinophagy markers LC3, while ferritinophagy inducer Rapa co-treatment triggered the degradation of LC3. Moreover, the results showed that DHQ notably inhibited degradation of FTH1 and NCOA4, but co-treatment with Rapa eliminated this effect (Figure 5D). Furthermore, The results also demonstrated that DHQ treatment significantly reduced the expression of α-SMA, collagen1 and fibronectin, whereas Rapa co-treatment dramatically increased the expression of MRC-5 activation makers (Figure 5E). Overall, these findings revealed that DHQ suppressed activated HBE cells ferroptosis by inhibiting ferritinophagy to alleviate MRC-5 activation *in vitro.*

## Discussion

Silicosis is one of the most prevalent types of pneumoconiosis caused by occupational exposure to respirable crystalline silica particles, which causes chronic lung inflammation, leading to progressive massive fibrosis and ultimately, respiratory failure (Leung et al., 2012). However, the underlying pathogenesis of silicosis is still unclear and effective therapies to halt disease progression or reverse established lung fibrosis are lacking. There is still an imperative need for more effective therapies. DHQ is an antifibrotic and antioxidant substance, that is, widely used. Guo et al. (2015) reported that DHQ prevented cardiac hypertrophy and delayed ventricular fibrosis when exposed to pressure overload. Moreover, Ding et al. (2018) showed that DHQ had nephroprotective effects, mainly reducing renal histopathological lesions in diabetic nephropathy by inhibiting ROS and NLRP3 inflammasome. Furthermore, Liu et al. (2021) demonstrated that DHQ inhibited inflammation, and attenuated CCl4-induced liver fibrosis by regulating PI3K/AKT/mTOR and TGF-β1/Smads pathways. Besides, Impellizzeri et al. (2015) discovered that DHQ ameliorated experimental pulmonary fibrosis induced by bleomycin. In addition, Unver et al. (2019) reported that DHQ protected the lung tissue from the toxic effects of cisplatin. These previous studies have confirmed that DHQ had anti-inflammatory and anti-fibrotic effects. The current study also demonstrated that DHQ prevented silica-induced features of lung fibrosis both *in vivo* and *in vitro*. (Figures 1, 2). Treatment with DHQ significantly reduced silica-induced the collagen deposition in C57/BL6 mice and MRC-5 cells compared to the SiO_2_ group. Additionally, DHQ treatment also significantly attenuated silica-induced augmentation of TGF-β expression in the C57/BL6 mice. TGF-β is a typical inducer in the process of fibrosis, promoting the differentiation of fibroblast and activating fibroblast to secrete excessive extracellular matrix.

Interestingly, existing data implied that cellular ferroptosis also has the potential to perform a key function in lung damage and pulmonary fibrosis (Li et al., 2019; Yoshida et al., 2019; Li Y. et al., 2020b; Liu P. et al., 2020b; Dong et al., 2020; Minagawa et al., 2020; Qiang et al., 2020; Tao et al., 2020). Li et al. (2019) discovered that the ferroptosis inhibitor improved radiation-induced lung fibrosis (RILF) *via* TGF-β1 down-modulation. Yoshida et al. (2019) demonstrated that CS-induced ferritinophagy and subsequent activation of ferroptosis played a unique function in COPD pathogenesis. Li Y. et al. (2020b) discovered that the inhibitor of apoptosis-stimulating protein of p53 suppressed ferroptosis and alleviated intestinal ischemia/reperfusion-induced acute lung damage. Dong et al. (2020) demonstrated that Nrf2 reduced ferroptosis and protected against acute lung damage caused by intestinal ischemia-reperfusion through regulating SLC7A11 and HO-1 expression. Liu P. et al. (2020b) reported that ferrostatin-1 alleviated lipopolysaccharide-induced acute lung injury *via* inhibiting ferroptosis. More interestingly, DHQ was identified as a potential ferroptosis inhibitor (Manigandan et al., 2015; Kuang et al., 2017; Wang W. et al., 2020b). Kuang et al. (2017) displayed that DHQ induced expression of nuclear factor erythroid 2-related factor 2 (Nrf2) and its downstream target genes to prevent ferroptosis in mouse skin epidermal JB6 P+ cells through epigenetic modifications. Manigandan et al. (2015) demonstrated that DHQ inhibited NF-κB-mediated wnt/β-catenin signaling *via* up-regulating Nrf2 pathway in experimental colon carcinogenesis. Wang W. et al. (2020b) verified that DHQ protected against renal fibrosis by activating the Nrf2 pathway. Dodson et al. (2019) reported that Nrf2 pathway played a critical role in mitigating lipid peroxidation and ferroptosis. Importantly, a growing body of studies showed that Nrf2 played a key role in ferroptosis-related pathways, including lipid metabolism and iron homeostasis (Huang et al., 2010; Sun et al., 2016; Kerins et al., 2018). These studies indicated that inhibition of ferroptosis had a significant protective effect against lung injury and fibrosis. In addition, DHQ might inhibited the activation of the ferroptosis by activating the Nrf2 pathway. However, the study of DHQ inhibiting HBE cells ferroptosis remained insufficient, and its potential molecular mechanism of protection was unclear. Similar to previous discoveries, our study confirmed that DHQ surely inhibited ferroptosis in C57BL/6 mice and HBE cells. Our results showed that the levels of ferroptosis markers GPX4 and GSH were significantly increased, and levels of iron, ROS, MDA, 4-HNE and fibrosis markers were reduced by DHQ treatment. These experimental findings supported that DHQ palyed an anti-fibrosis role by inhibiting the ferroptosis in activated HBE cells.

Ferritinophagy, which was defined as the process of degrading ferritin and releasing iron, was implicated in the pathophysiologic process of iron metabolism (Baksi and Singh, 2017; Masaldan et al., 2018; Li N. et al., 2020a; Jiang et al., 2020). Li N. et al. (2020a) discovered that NCOA4 could directly interacted with ferritin and degraded it in a ferritinophagy-dependent way, resulting in the release of a significant quantity of iron. Additionally, Masaldan et al. (2018) discovered that iron accumulation in senescent cells was associated with decreased ferritinophagy and suppression of ferroptosis. Besides, Jiang et al. (2020) found that a high-fat diet may inhibited ferritinophagy and disrupted iron metabolism, leading to hepatic insulin struggle through endoplasmic reticulum stress. Moreover, Baksi et al. demonstrated that α-synuclein impaired ferritinophagy, resulting in the accumulation of iron-rich ferritin in the outer retina *in vivo* and retinal-pigment-epithelial (RPE) cells *in vitro* (Baksi and Singh, 2017). Currently, accumulating reports showed that ferritinophagy was the upstream pathway of ferroptosis (Mancias et al., 2014; Gao et al., 2016; Latunde-Dada, 2017; Masaldan et al., 2018; Zhang et al., 2018). These studies demonstrated that ferritinophagy was one of the important processes for ferroptosis. Consistent with previous literature, our further experimental data also testified that ferritinophagy was involved in ferroptosis inhibition by DHQ in activated HBE cells. Firstly, the expression of ferritinophagy markers LC3, FTH1, and NCOA4 were detected. The results showed that DHQ suppressed ferroptosis in activated HBE cells *via* inhibiting ferritinophagy. In contrast, ferritinophagy inducer rapamycin significantly reversed the effect of ferroptosis inhibition of DHQ.

## Conclusion

**FIGURE 6:**
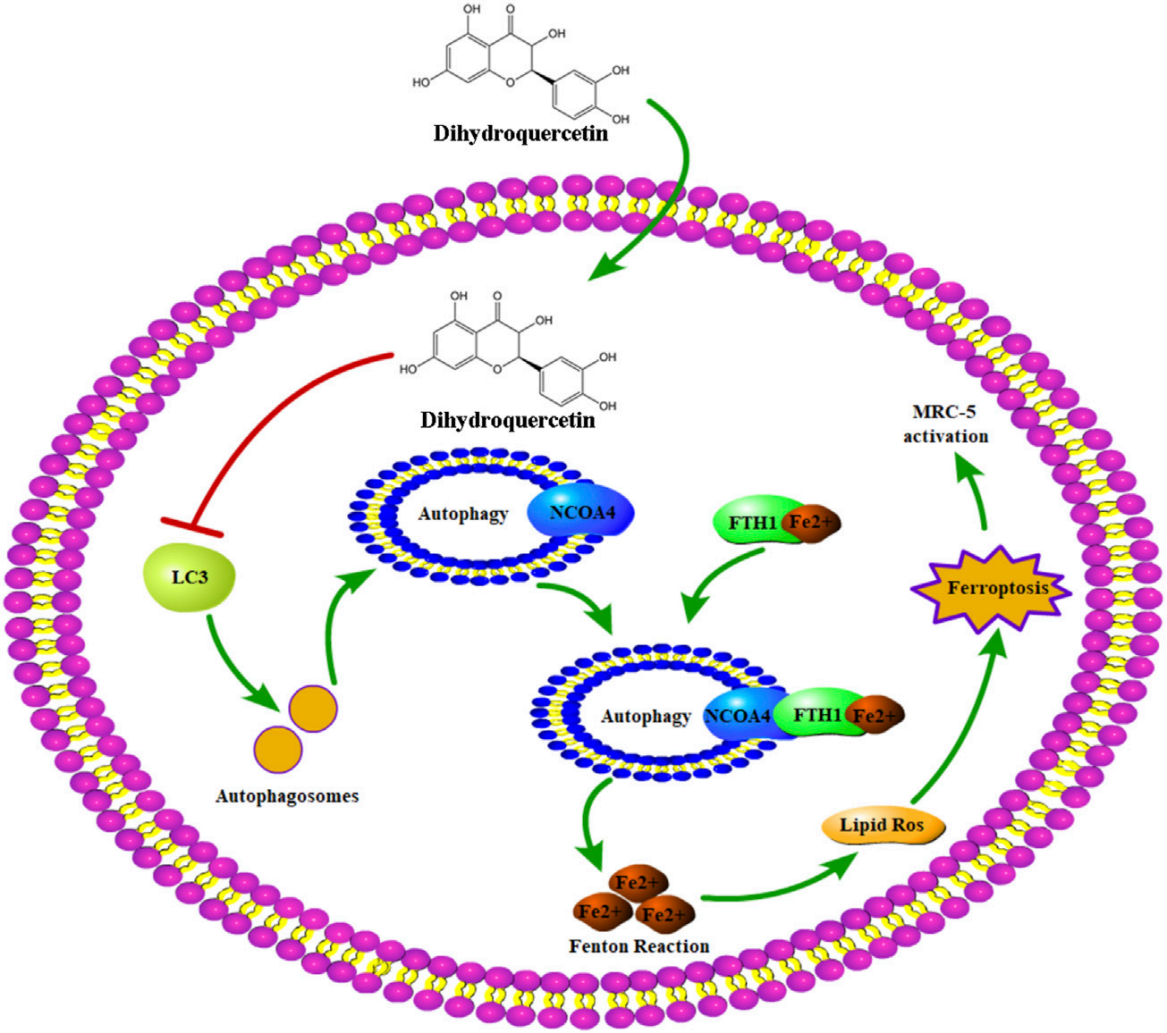
The underlying mechanism of DHQ against silica-induced lung fibrosis.

In summary, this study showed the protective effect of DHQ against silica-induced features of pulmonary fibrosis *in vivo* and *in vitro* and further identified the important role of ferritinophagy-mediated ferroptosis pathway in the anti-fibrosis mechanism of DHQ (Figure 6). These findings revealed part of mechanism for understanding the protective effect of DHQ against silica-induced pulmonary fibrosis, and provided a theoretical basis for the clinical application of DHQ in silicosis. However, this study also had limitations. Whether DHQ might play a protective role under other types of pneumoconiosis is unknown. Similarly, it is not clear whether DHQ could inhibit ferritinophagy to alleviate lung fibrosis *in vivo*, which is also worthy of further exploration. Furthermore, whether DHQ could play an effective therapy for silicosis patients is also unclear. Therefore, we will continue to study the full anti-fibrotic potential of DHQ. Kerins and Ooi, 2018, Latunde-Dada, 2017, Sun et al., 2016.

## Data Availability

The original contributions presented in the study are included in the article/Supplementary Material, further inquiries can be directed to the corresponding authors.
