# Supplementary material for: Dihydroquercetin Attenuates Silica-Induced Pulmonary Fibrosis by Inhibiting Ferroptosis Signaling Pathway
Source: Front Pharmacol. 2022 May 12;13:845600. doi: 10.3389/fphar.2022.845600 (PMC9133504; doi:10.3389/fphar.2022.845600)
Supplement: Supplementary file 1 [file Table1.DOCX]

Table S1 Primer sequences

| Gene | Forward | Reverse |
| --- | --- | --- |
| Collagen I | 5'-CGGAGGAGAGTCAGGAAGG-3' | 5'-CACAAGGAACAGAACAGAACA-3' |
| Fibronectin | 5'-CACTTACCGAGTGGGTGACACTT-3' | 5'-GCAGGTACAGTCCCAGATCATG-3' |
| α-SMA | 5'-AAGAAGAGGACAGCACT-3' | 5'-TCCCATTCCCACCATCAC-3' |
| GAPDH | 5'-CCATGAGAAGTATGACAACAGCC -3' | 5'-GGGTGCTAAGCAGTTGGTG-3' |
